# Comparison and optimization of protein extraction and two-dimensional gel electrophoresis protocols for liverworts

**DOI:** 10.1186/s13104-020-4929-1

**Published:** 2020-02-07

**Authors:** Sandhya Yadav, Akanksha Srivastava, Subhankar Biswas, Neha Chaurasia, Sushil Kumar Singh, Sanjiv Kumar, Vaibhav Srivastava, Yogesh Mishra

**Affiliations:** 1 Department of Botany, Centre of Advanced Study in Botany, Institute of Science, Banaras Hindu University, Varanasi, 221005 India; 2 Department of Biotechnology and Bioinformatics, North Eastern Hill University, Shillong, 793022 India; 3 Botanical Survey of India Northern Regional Centre, 192, Kaulagarh Road, Dehradun, Uttarakhand 248003 India; 4 Division of Glycoscience, Department of Chemistry, School of Engineering Sciences in Chemistry, Biotechnology and Health, Royal Institute of Technology (KTH), AlbaNova University Centre, 10691 Stockholm, Sweden

**Keywords:** *Dumortiera hirsuta*, Liverworts, *Marchantia paleacea*, *Plagiochasma appendiculatum*, Proteomics, Two-dimensional gel electrophoresis (2-DE)

## Abstract

**Objective:**

Liverworts possess historical adaptive strategies for abiotic stresses because they were the first plants that shifted from water to land. Proteomics is a state-of-the-art technique that can capture snapshots of events occurring at the protein level in many organisms. Herein, we highlight the comparison and optimization of an effective protein extraction and precipitation protocol for two-dimensional gel electrophoresis (2-DE) of liverworts.

**Results:**

We compared three different protein extraction methods, i.e.,1.5 M Tris–HCl (pH 8.8), 50 mM Tris–HCl (pH 7.5), and polyvinylpolypyrrolidone (PVPP) extraction, followed by three precipitation methods, i.e., 80% ethanol, 80% acetone, and 20% tricholoroacetic acid (TCA)–acetone, in a liverwort *Dumortiera hirsuta*. Among these methods, 50 mM Tris–HCl (pH 7.5) extraction, followed by 20% TCA–acetone precipitation, appeared to be more suitable for 2-DE. Furthermore, we performed modifications during protein washing, re-solubilization in rehydration buffer and isoelectric focusing (IEF). The modifications provided us better results in terms of protein yield, resolution, spot numbers, and intensities for 2-DE gels of *D. hirsuta* and other two liverworts, i.e., *Marchantia paleacea* and *Plagiochasma appendiculatum*. Furthermore, we randomly selected spots from the 2-DE gel of *D. hirsuta* and identified using mass spectrometry, which confirms the applicability of this protocol for liverworts proteomics.

## Introduction

Two-dimensional gel electrophoresis (2-DE) coupled with mass spectrometry is a classical approach for quantitatively analyzing protein amounts in complex extracts. Despite having certain technological limitations in terms of its throughput and analyzable protein ranges, this technique is generally used to tackle various biological questions related to stress tolerance/adaptation [1, 2].

2-DE has been extensively applied to several organisms for identifying proteins and their expression patterns under certain experimental conditions [1, 3, 4]. To obtain good 2-DE gels, the presence of a suitable method of protein extraction, precipitations, and solubilization is important, and these steps require extra consideration if we are planning for plant proteomics. Generally, plant tissues contain numerous organic acids, phenolic compounds, lipids, carbohydrates, pigments, and proteases, which interfere with protein isolation and separation processes [5]. Over the past two decades, considerable effort has been made to get rid of interfering agents that eventually had enhanced plant proteomic analysis [5–7]. However, compared to other plant species, there are very few proteomic studies for bryophytes.

Bryophytes were the earliest land plants that conquered land for the first time more than 500 million years ago [8]. Considering the evolutionary importance of bryophytes, there is an urgent requirement to study their adaptabilities in natural habitats at the protein level. In the past two decades, few bryophytes, namely, *Physcomitrella patens* [8] and *Marchantia polymorpha* [9], have been developed as model organisms, which promoted plant development and genomic studies. Unfortunately, very few proteomic studies have been conducted for bryophytes [10–12].

Among bryophytes, liverworts, being a primitive class, are the pioneers of terrestrialization and still possess few characteristics of aquatic life [13]. The proteomic study of liverworts may uncover those proteins that might have helped them in the process of terrestrialization and may provide an insight into the evolutionary mechanisms that have contributed to the complexity of modern land plants.

Liverworts produce a number of secondary metabolites, such as terpenoids, phenolics, sterols, tannins, flavonoids, and aromatic compounds, to protect themselves from various herbivores, pathogenic microorganisms, and abiotic stresses [14, 15]. In addition to these secondary metabolites, liverwort contains some unique compounds such as sesquiterpenoids of pinguisane and dumortane-type, and sacculatane-type, neodenudatane-type and ent-verticillane-type diterpenoids that were only found in liverworts [16–18]. While performing the proteomic analysis of liverworts, these compounds co-precipitate with proteins and hinder isoelectric focusing (IEF) that eventually results in the streaking and smearing of 2-DE gels [5]. Therefore, it is very important to optimize an efficient protocol that could overcome these constrains so that better-resolved 2-DE gels of liverworts can be produced.

Considering the evolutionary significances of liverworts and the lack of proteomic information, this study deals with the comparison and optimization of protein extraction, followed by a precipitation step before gel-based proteomic analysis. To our knowledge, this is the first study examining the proteomics of liverworts. Here, we compared the different procedures for the extraction, precipitation, and evaluation of soluble proteins and 2-DE profiles of selected liverworts, namely, *Dumortiera hirsuta*, *Plagiochasma appendiculatum* and *Marchantia paleacea*.

## Main text

### Methods

#### Plant materials and experimental design

**Fig. 1:**
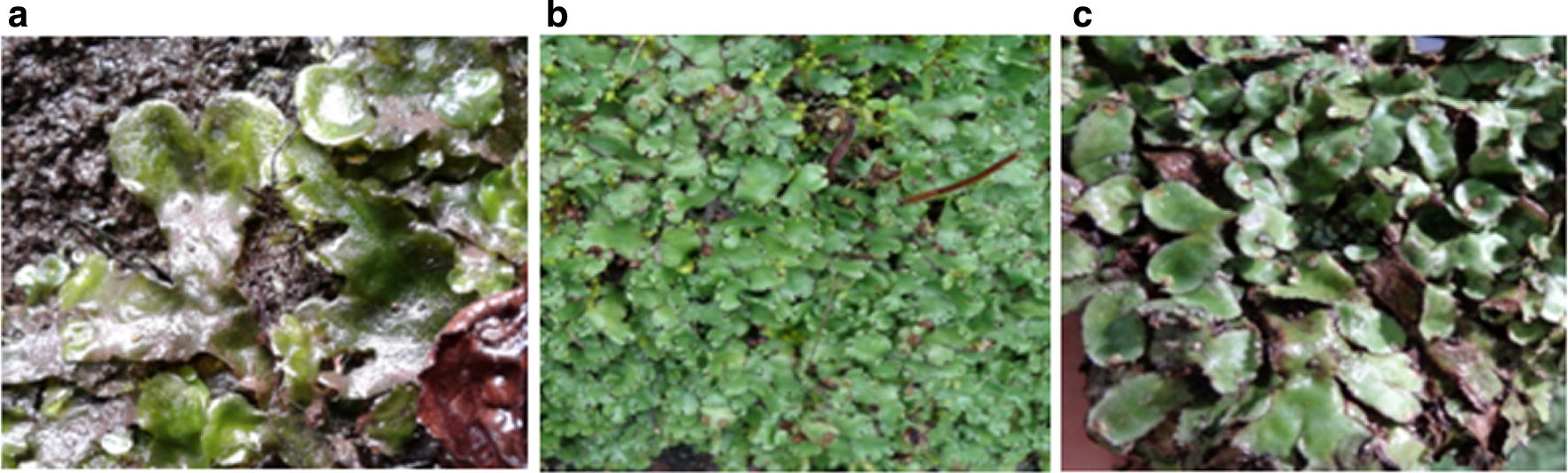
Images of the gametophytes of liverworts in their natural habitat: **a***D. hirsuta,***b***M. paleacea*, and **c***P. appendiculatum*

Three representative liverworts, *D. hirsuta*, *M. paleacea*, and *P. appendiculatum* (Fig. 1), were collected from Shillong (Meghalaya), India, at a latitude of 25.5788°N and longitude of 91.8933°E. For sample collection, thalli were washed with distilled water to remove the soil and other impurities and immediately frozen in liquid nitrogen and brought to the laboratory.

We initiated this work by comparing three well known plant protein extraction buffers in *D. hirsuta* followed by protein precipitation in three different organic solvents. Once the protein extraction buffer and precipitation method was finalized, we performed 2-DE for *D. hirsuta* with certain modifications. Then, we applied the same protocol to two other liverworts namely *M. paleacea*, and *P. appendiculatum.* All experiments were conducted in triplicate (at both biological and technical level).

#### Selection of buffers, protein quantifications and SDS-PAGE

Three well-known plant protein extraction buffers, i.e., (i) 50 mM Tris–HCl (pH 7.5), 100 mM KCl and 10% glycerol [19], (ii) 1.5 M Tris–HCl (pH 8.8) [20] and (iii) polyvinylpolypyrrolidone (PVPP) extraction buffer containing (0.2 M 3-(*N*-morpholino) propanesulfonic acid (MOPS) pH 7.0, 5% PVPP, 1% triton X-100, 10% glycerol, and 2 mM DTT; [21], were tested for isolating the cytosolic proteins from three liverworts. Additional details on protein quantification and SDS-PAGE analysis are provided in Additional file 1.

#### Comparison of protein precipitation methods and optimization of 2-DE

As per the literature, three different methods of protein precipitations were tested in the case of *D. hirsuta* namely, ice-chilled 80% ethanol [22], 80% acetone [20], and TCA–acetone (with varied concentration of TCA, i.e., 10%, 15%, and 20%) [23]. Moreover, additional applications and comparison of these precipitation methods are described in Additional file 1. In addition to these steps regarding 2-DE optimisation, spot excision, in-gel tryptic digestion, and mass spectrometry of *D. hirsuta* are illustrated in Additional file 1.

#### Optimized method of 2-DE for *D. hirsuta* was applied in the remaining two liverworts

Once the 2-DE method was optimized for *D. hirsuta*, the same protocol was applied to *M. paleacea* and *P. appendiculatum*.

### Results

**Fig. 2:**
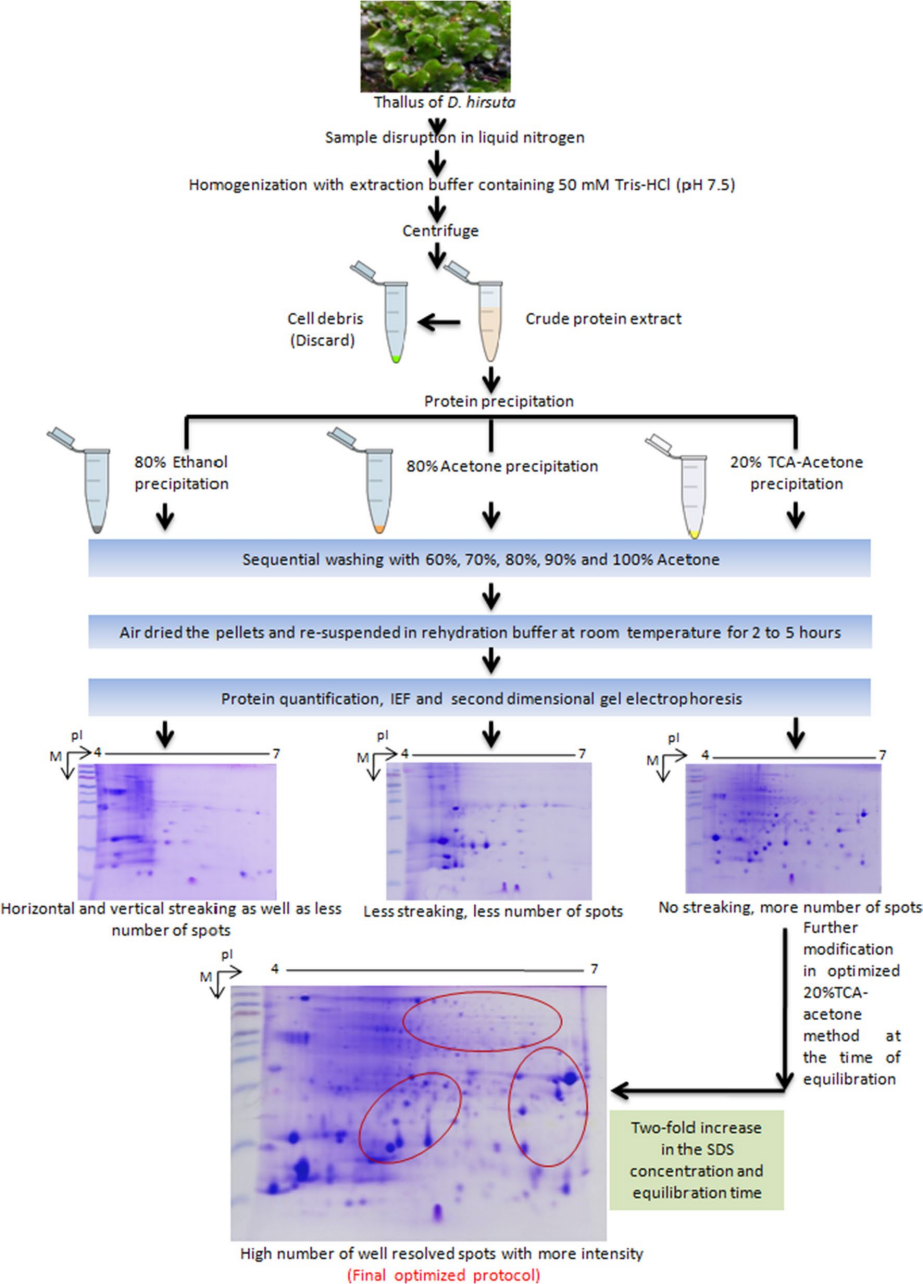
Flow chart of the optimization process of proteins precipitation and 2-DE gel separation in *D. hirsuta*. The red-marked circles on 2-DE gels show novel well-resolved spots

In this study, three distinctive protein extraction buffers, i.e., 50 mM Tris–HCl (pH 7.5), 1.5 M Tris–HCl (pH 8.8), and PVPP-containing buffer, were evaluated in *D. hirsuta* in terms of protein yield and better resolution on SDS-PAGE. Among them, 50 mM Tris–HCl (pH 7.5) showed a high protein yield and better resolved SDS-PAGE (Additional file 2: Figures S1, S2). Furthermore, 50 mM Tris–HCl (pH 7.5) extracted proteins were subjected to three different protein precipitation method, i.e., 80% ethanol, 80% acetone, and 20% TCA–acetone. The result demonstrated that the 20% TCA–acetone precipitation method with slight modification such as concentration gradient acetone washing of protein samples, increase in incubation of protein pellets in the rehydration buffer, slight alteration in IEF program and a twofold increase in the SDS concentration and equilibration time, produced better results for the proteomic study, which is reflected in terms of more number of protein spots on 2-DE gels with lesser streaking and smearing on gel (Additional file 2: Figures S5, S6). The abovementioned results could be attributed to TCA effectively concentrating the proteins by removing a majority of secondary metabolites (phenolics, terpenoids, and pigments) from the precipitated proteins. The entire optimization process has been shown in the form of a flow chart (Fig. 2). Furthermore, we excised randomly selected spots from the 2-DE gel of *D. hirsuta*, which were successfully identified using mass spectrometry. The details of the above-mentioned results are provided in Additional file 2: Figures S1–10.

**Fig. 3:**
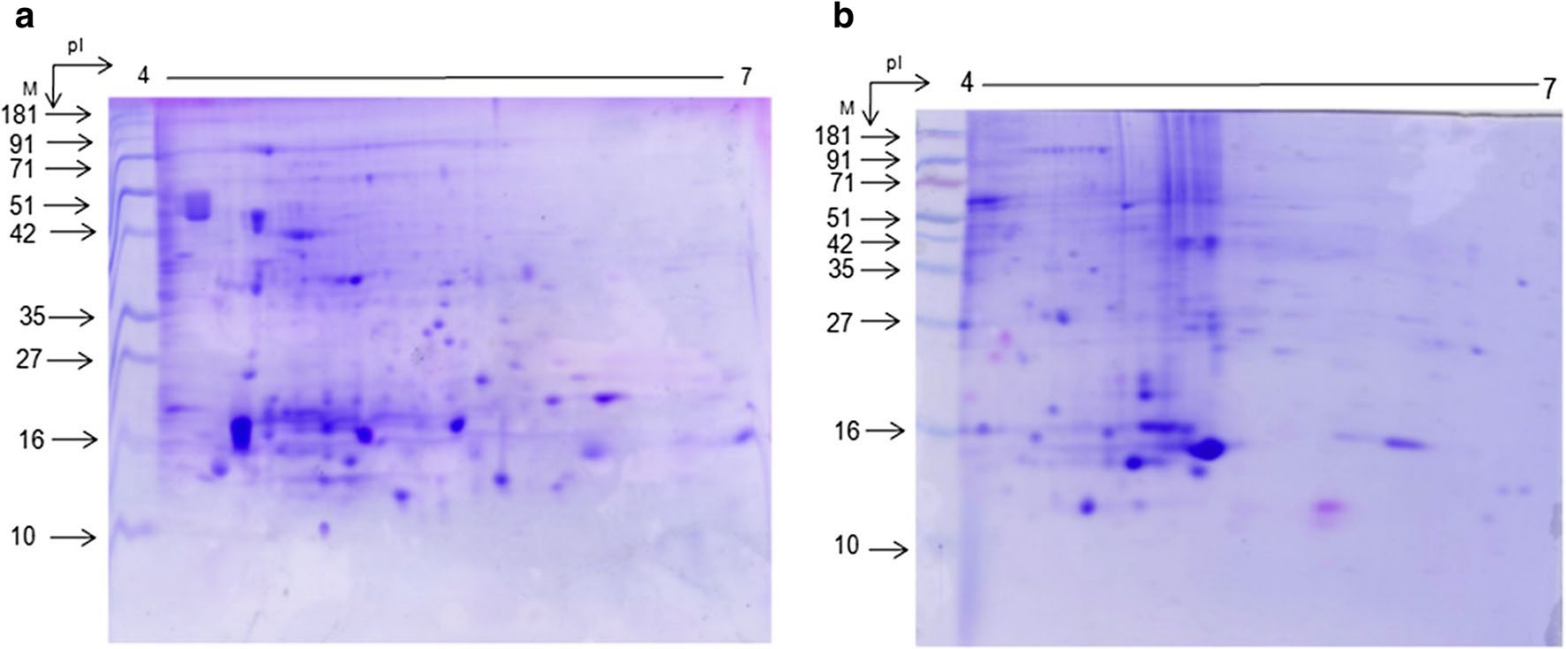
2-DE gel profiles of **a***M. palaceae* and **b***P. appendiculatum* using modified 50 mM Tris–HCl (pH 7.5) extraction, followed by 20% TCA–acetone precipitation method

To confirm the applicability of this method, it was applied to another two selected liverworts in which well-resolved 2-DE gels were observed (Fig. 3).

### Discussion

Liverworts produce a wide array of secondary metabolites; few of these compounds are unique to liverworts and have not been reported in any other plants, fungi, and marine organisms [15, 24]. During protein extraction, these secondary metabolites form very strong hydrogen bonds with proteins and build irreversible complexes that eventually result in the streaking and smearing of 2-DE gels [25]. Therefore, to obtain high-quality protein samples that can offer a satisfactory 2-DE gel, the removal of these interfering compounds are essential. Generally, two strategies are used to remove the interfering compounds from extracted proteins. In first strategy, contaminants are removed before protein extraction; however, in second strategy, removal is performed during and after protein extraction [5]. In this study, we focused on second strategy in which we compared three different protein extraction buffers, namely, 1.5 M Tris–HCl (pH 8.8), 50 mM Tris–HCl (pH 7.5), and PVPP extraction buffer, in terms of both protein yield and better separation in SDS-PAGE of *D. hirsuta*. In this study, PVPP extraction buffer improved the protein yield in crude extract; however, after precipitation in 20% TCA–acetone, the amount of soluble protein was found to be lesser compared to 50 mM Tris–HCl (pH 7.5) and 1.5 M Tris–HCl (pH 8.8) (Additional file 2: Figure S1A, B). After precipitation and resolubilization in the rehydration buffer, 50 mM Tris–HCl (pH 7.5) showed a higher protein yield among all tested buffers. Furthermore, the SDS-PAGE analysis showed a better band profile for proteins extracted using 50 mM Tris–HCl (pH 7.5) compared to others. Based on these results, we concluded that the addition of PVPP neither improved the amount of protein in the precipitated samples of *D. hirsuta* nor showed better separation of proteins in SDS-PAGE; therefore, this procedure was eliminated from further study. Furthermore, similar results have been reported in case of mosses because PVPP addition did not improve the protein content and quality of gel [20]. The success of a simple but an efficient buffer, i.e., 50 mM Tris–HCl 7.5, can be probably attributed to it providing a very favourable condition for the solubility and stability of multiple proteins. Previously, this buffer has been used to obtain better quality of 2-DE gels in mosses [26].

After obtaining improved results for protein extraction in 50 mM Tris–HCl (pH 7.5) buffer, we focused on evaluating protein precipitation methods. We used three organic solvents for precipitation, namely, 80% ethanol, 80% acetone, and TCA–acetone (in the range of 10%, 15%, and 20% TCA). Note that protein pellets obtained using 20% TCA–acetone were creamy white in color and offered good quality of 2-DE gels in terms of lack of streaking, numbers, and distribution of spots and resolution of gels (Additional file 2: Figure S5 A–E). Furthermore, a creamy white color of protein pellet indicates that a high amount of TCA was very effective for removing a majority of secondary metabolites. Moreover, TCA–acetone precipitation was advantageous because it removed interfering compounds and simultaneously inactivated components involved in the degradation and modification of proteins such as proteases, phenoloxidases, and peroxidases [20, 23, 27]. There are reports of various types of plants in which TCA–acetone precipitation has been successfully used for protein precipitation followed by 2-DE [28–30]. However, few contaminants occasionally get co-precipitated with proteins, which affect both the quality and solubility of proteins. To eliminate these contaminants and increase the quality and solubility of proteins, we performed certain modifications, i.e. (i) sequential concentration gradient acetone washing of protein pellets, (ii) prolonged incubation of protein pellets in rehydration buffer, (iii) alteration in IEF program, (iv) two-fold increase in the SDS concentration in equilibration buffer, and (v) increase in SDS-equilibration time of IPG strips. Based on our results, these amendments facilitated non-protein contaminant removal and protein re-dissolution, which ultimately increased the number of protein spots, reduced the horizontal and vertical streaking, and enhanced the resolution of 2-DE gels in the case of *D. hirsuta* (Additional file 2: Figure S6). All protein spots selected for mass spectrometry resulted in successful identification, which indicates the compatibility of optimized method using mass spectrometry and its reliability for downstream processing. Furthermore, a similar protocol was successfully applied for the remaining two liverworts, namely, *M. paleacea* and *P. appendiculatum*, which was confirmed by better-resolved 2-DE gels (Fig. 3).

Currently, phenol-based methods have attracted considerable attention and are commonly used in plant proteomics. In this study, we attempted phenol-based protein extraction, followed by ammonium acetate–methanol precipitation as described in *P. patens* [31]. Note that a phenol-based method was not better suited for liverworts because the color of precipitated proteins (indicating contaminations) and acquired 2-DE gel was not found as satisfactorily as those produced by 50 mM Tris–HCl (pH 7.5) extraction, followed by 20% TCA–acetone precipitation (Additional file 2: Figure S10).

The greatest advantage of the modified protocol for protein extraction and precipitation is that the method is very simple and fast; therefore, it can be applied to study the proteome of a wide range of liverworts.

### Limitations

Despite being the first land plants and having a long evolutionary history, there have been very few proteomic studies in liverworts. This could be attributed to the presence of enormous secondary metabolites, which hinders the IEF and does not let the normal 2-DE protocol to be directly applied. Therefore, it is very important to optimize an efficient protocol for gel-based proteomics of liverworts.

## Supplementary information

**Additional file 1.** Methods in detail.

**Additional file 2.** Additional results.

**Additional file 3: Table S1.** List of identified proteins from the 2-DE gel of *D. hirsuta* using mass spectrometry.

## Data Availability

All data generated or analyzed during this study are included in this published articles [and in Additional files 1, 2, 3].

## Abbreviations

2-DE: Two dimensional electrophoresis
ANOVA: One-way analysis of variance
BPB: Bromophenol blue
CHAPS: 3-[(3-cholamidopropyl) dimethylammonio]-1-propanesulfonate
DTT: Dithiothreitol
IEF: Isoelectric focusing
IPG: Immobilized pH gradient
MOPS: 3-(*N*-morpholino) propanesulfonicacid
MS: Mass spectrometry
PVPP: Polyvinylpolypyrrolidone
SDS-PAGE: Sodium dodecyl sulphate polyacrylamide gel electrophoresis
TCA: Trichloroacetic acid
TEMED: N,N,N′,N′-Tetramethyl ethylenediamine
Tris: Tris (hydroxymethyl) aminomethane
