# Supplementary material for: Comparison and optimization of protein extraction and two-dimensional gel electrophoresis protocols for liverworts
Source: BMC Res Notes. 2020 Feb 7;13:60. doi: 10.1186/s13104-020-4929-1 (PMC7006083; doi:10.1186/s13104-020-4929-1)
Supplement: Supplementary file 1 — Additional file 1. Methods in detail. [file 13104_2020_4929_MOESM1_ESM.docx]

**Additional file 1**

**Comparison and optimization of protein extraction and two-dimensional gel electrophoresis protocols for liverworts**

Sandhya Yadav^1^, Akanksha Srivastava^1^, Subhankar Biswas^1^, Neha Chaurasia^2^, Sushil Kumar Singh^3^, Sanjiv Kumar^4^, Vaibhav Srivastava^4^**^*^**, Yogesh Mishra^1^**^*^**

**Affiliations:**

*^1^Department of Botany, Centre of Advanced Study in Botany, Institute of Science, Banaras Hindu University, Varanasi-221005, India*

*^2^Department of Biotechnology and Bioinformatics, North Eastern Hill University,
Shillong- 793022, India*

*^3^Botanical Survey of India Northern Regional Centre, 192, Kaulagarh Road, Dehradun
Uttarakhand, 248003*, *India*

*^4^Division of Glycoscience, Department of Chemistry, School of Engineering Sciences in Chemistry, Biotechnology and Health, Royal Institute of Technology (KTH), AlbaNova University Centre, Stockholm,10691, Sweden*

***Corresponding Authors**: Vaibhav Srivastava (vasri@kth.se); Yogesh Mishra (ymishra@bhu.ac.in)

**Methods**

**Comparison of protein extraction buffers**

Three well-known plant protein extraction buffers, i.e., (i) 50 mM Tris-HCl (pH 7.5) buffer containing 50 mM Tris-HCl, 100 mM KCl and 10% glycerol [1], (ii) 1.5 M Tris-HCl (pH 8.8) [2] and (iii) Polyvinylpolypyrrolidone (PVPP) extraction buffer containing (0.2 M 3-(N-morpholino) propanesulfonicacid (MOPS) pH 7.0, 5% PVPP, 1% triton X-100, 10% glycerol, and 2 mM DTT; [3], were tested for isolating the cytosolic proteins from three liverworts. Initially, we cleaned 2.5 g of thalli and ground them under liquid nitrogen using a mortar and pestle. The samples were then mixed in 5 ml of these three buffers along with the cocktail of protease inhibitor (Sigma). To obtain the soluble fraction of proteins, the extracts were centrifuged at 12000×g (4°C) for 1 h. afterwards the supernatant was transferred to clean tubes for subsequent experiments. Apart from above mentioned protein extraction buffers, we also tested the well-known protein extraction method based on Tris-saturated phenol pH 7.5 [4] for *D. hirsuta* (Additional file [2](https://proteomesci.biomedcentral.com/articles/10.1186/s12953-019-0149-9#MOESM1): Figure S10). However, it was not used for further proteomic study as discussed later.

**Protein quantification**

Total cell protein (mg ml^-1^) obtained using the aforementioned buffers was estimated by Bradford’s (1976) [5] method before and after 20% TCA-acetone protein precipitation as it showed a better resolution among the tested protein precipitation methods (described in coming section). Briefly, 5 µL of protein sample was mixed into 1 ml of Bradford reagent (BioRad Protein, Assay) and incubated for 5 min at RT. Absorbance at 595 nm was measured with the help of  a Spectrophotometer (UV-VIS Spectrophotometer, Shimadzu , Japan). For equal loading of the protein samples on each IPG strip,  air-dried protein pellets were re-suspended in rehydration buffer containing 7 M urea, 2 M thiourea, 4% CHAPS, 40 mM DTT, and 1.0% IPG buffer)  and quantified using Bradford’s assay as described above. It is worth mentioning that the final concentration of urea in the Bradford reagent was 0.035 M, which is significantly very less than its concentration limit (3M) mentioned by the manufacturer for Bradford assay. ​

**SDS-PAGE**

For SDS-PAGE, we have loaded 25 µg protein on each well, which was isolated using 50 mM of Tris-HCl (pH 7.5), 1.5 M of Tris-HCl (pH 8.8) and PVPP extraction buffer, respectively. Before loading on each well protein samples were mixed in 1X SDS gel*-*loading buffer (50 mM Tris-HCl pH 6.8, 2% SDS, 10% (V/V) glycerol, 100 mM DTT, and 0.1% bromophenol blue (BPB)) and loaded on gel (5% stacking and 15% resolving) after heating at 95°C for ̴ 5 min. The samples were then run initially at 80 V for 1 h, and then the voltage was increased to 100 V, and the gels were run on the same current till the tracking dye crossed the gels using a vertical Bio-Rad mini-Protean Tetra Cell.

**Comparison of protein precipitation methods and optimization of 2-DE**

As per the literature, three different methods of protein precipitations were tested in the case of *D. hirsuta*. Ice-chilled 80% ethanol [6], 80% acetone [2], and TCA-acetone [7] were selected to precipitate the proteins (with varied concentration of TCA, i.e., 10%, 15%, and 20%). The total cell proteins obtained by 50 mM Tris-HCl (pH 7.5) were precipitated with six volumes of these three solvents, which were left overnight at −20°C and centrifuged at 8000×g for 15 min. The precipitated proteins were subjected to a concentration gradient-chilled acetone washing with 60%, 70%, 80%, and 90% acetone. They were then finally washed with 100% acetone by mixing the proteins pellets with a micropipette. This gradient washing helped with removal of extra salt and other interfering compounds from the precipitated protein. Air-dried protein pellets were re-suspended in rehydration buffer containing 7 M urea, 2 M thiourea, 4% CHAPS, 40 mM DTT, and 1.0% IPG buffer) and left at room temperature for 4 h while, occasional mixing and quantified before 2-DE. After that IPG buffer (suitable pI range) and traces of BPB was added to solubilised protein. Then, this mixture was centrifuged at 12,000×g for 10 min at room temperature to remove insoluble particles that can clog the gel pores. A total of 250 μl of rehydration buffer containing 500 μg proteins was applied to the IPG strips (pH 3–10; pH 4–7; 13 cm; GE healthcare, USA), and the strips were covered with mineral oil overnight at room temperature for rehydration. Isoelectric focusing (IEF) was conducted at 20°C with EttanIPGphor system (GE Healthcare, USA) in five steps: 50 V for 3 h, 500 V for 1 h, 1000 V for 1 h, 8000 V for 3 h, and 8000 V for 1 h with a maximum allowed current of 50 μA. The focused IPG strips were equilibrated by incubating in first equilibration buffer containing 6 M urea, 30% glycerol, 4% SDS, 50 mM Tris–HCl at pH 8.8 and 1% DTT with a pinch of BPB for 45 min, followed by incubation in the second equilibration buffer containing 2.5% iodoacetamide in the first equilibration solution rather than 1% DTT for 45 min. The strips were then rinsed with tris-glycine electrophoresis buffer and placed on the surface of 15.0 % SDS-PAGE. We laid 0.8% of melted agarose on the strip to minimize the displacement. We then carried out electrophoresis at 125 V till the dye front reached the gel’s lower bottom.

**Optimized method of 2-DE for *D. hirsuta* was applied in the remaining two liverworts**

Once the 2-DE method was optimized in case of *D. hirsuta*, the same protocol was applied for *M. paleacea* and *P. appendiculatum.* First, the cytosolic proteins were extracted in 50 mM Tris-HCl (pH 7.5) buffer and precipitated in 20% TCA-acetone. The precipitated proteins were then solubilized in rehydration buffer and 500 μg of protein for each samples were applied on IPG strips. We then performed equilibration and second dimension steps.

**Coomassie staining and destaining**

The SDS-PAGE and 2D-PAGE gels were stained with PhastGel® blue R and PlusOne Coomassie tablets, and then destained by 10% acetic acid. Note that the destained gels were scanned and saved for further analysis.

**Analysis of gels**

Coomassie-stained gel of SDS-PAGE was analysed using ImageQuant LAS 500 software (GE Healthcare). We used PDQuest™ Basic version 8.0.1 (Bio-Rad) to analyze 2-DE gels of *D. hirsuta* obtained using different protein precipitation methods. After background subtraction, the spots were automatically detected using a Spot Detection Parameter Wizard based on a Gaussian model with standardized parameters. Comparison between spot quantities across the gels was performed and normalization was performed using a local regression model. Only the protein spots of low and high intensities from the finest gel of *D. hirsuta* were randomly selected for mass spectrometry analysis.

**In-gel tryptic digestion and mass spectrometry**

The protein spots of interest were manually excised from the gel and processed for mass spectrometry analysis. The excised protein gel spots were incubated in 0.2 ml of reagent 1 (35% acetonitrile in 20 mM ammonium bicarbonate) for 30 min at room temperature. This step was repeated twice to completely destain the samples. The gel samples were then dehydrated for 5 min in 0.2 ml of 100% acetonitrile. After incubation, acetonitrile was discarded and gel pieces were dried in a speed vac (Eppendorf, Germany). Gel pieces were rehydrated by adding 20 µl of trypsin (10 ng/ µl) and incubated on ice for 45 min. After removing excess trypsin, we added 30mMof ammonium bicarbonate to cover the hydrated gel pieces and incubated at 37°C for 16 h. The resulting peptides were extracted, dried, and re-dissolved in 0.1% formic acid for mass spectrometry analysis [8]. We performed peptide analysis by reversed-phase liquid chromatography electrospray ionization mass spectrometer (LC-ESI-MS/MS) using a nanoACQUITY ultra-performance liquid chromatography (UPLC) system coupled to a Q-TOF mass spectrometer (Xevo Q-TOF; Waters, Milford, MA, USA) [9]. We processed the resulting MS raw data files using Mascot Distiller (version 2.4.3.2, Matrix Science, London, UK), and the resulting files were submitted to a local Mascot (Matrix Science, version 2.3.1) server using the protein database of *M. polymorpha* (24,674 sequences; 1,09,38,663 residues). The following settings were used for the database search: trypsin-specific digestion with two missed cleavage allowed; carbamidomethylatedcysteine as fixed and oxidized methionine as variable modifications; peptide tolerance of 100 ppm; and fragment tolerance of 0.2 Da. Peptides with Mascot ion scores exceeding the threshold for statistical significance (p<0.05) were selected.

Statistics of protein properties (number of amino acids, molecular weights, and pI) were calculated using pepstats (EMBOSS v6.6.0.0) [10]. Conserved domain(s) in the proteins were identified using Conserved Domains Database (CDD).Functional annotation of the proteins was performed using Blast2GO [11].

**Statistical analysis**

All the experiments were performed in triplicates. To determine the significant differences in protein quantification, one–way analysis of variance (ANOVA) was applied using SPSS 16.0 software. When ANOVA was significant, Tukey’s post hoc comparison was performed considering a confidence level of 95 percent (P < 0.05).

**Additional file 1 references:**

1. Klose J, Kobalz U. Two dimensional electrophoresis of proteins: an updated protocol and implications for a functional analysis of the genome. Electrophoresis. 1995;16(1):1034-1059

2. Barbara C, Braglia R, Basile A, Cobianchi RC, Forni C. Proteomics and Bryophytes: a comparison between different methods of protein extraction to study protein synthesis in the aquatic moss *Leptodictyum riparium* (Hedw.). Caryologia. 2007;60(1-2):102-105.

3. Laing W, Christeller J. Extraction of proteins from plant tissues. Curr Protoc Protein Sci. 2004;38(1):4-7.

4. Wang X, Yang P, Gao Q, Liu X, Kuang T, Shen S, et al. Proteomic analysis of the response to high-salinity stress in *Physcomitrella patens*. Planta. 2008;228(1):167-177.

5. Bradford MM. A rapid and sensitive method for the quantification of microgram quantity of proteins utilising the principle of protein dye binding. Anal Biochem. 1976;72(1-2):248-254.

6. Hansson SF, Puchades M, Blennow K, Sjögren, M, Davidsson, P. Validation of a pre fractionation method followed by two-dimensional electrophoresis–Applied to cerebrospinal fluid proteins from frontotemporal dementia patients. Proteome Sci. 2004;2(1):7.

7. Damerval C, DeVienne D, Zivy M, Thiellement H. Technical improvements in two dimensional electrophoresis increase the level of genetic variation detected in wheat seedling proteins. Electrophoresis.1986;7(1):52-54.

8. Srivastava V, Obudulu O, Bygdell J, Löfstedt T, Rydén P, Nilsson R, et al. OnPLS integration of transcriptomic, proteomic and metabolomic data shows multi-level oxidative stress responses in the cambium of transgenic hipI-superoxide dismutase Populus plants. BMC Genomics. 2013;14(1):893.

9. Srivastava V, Rezinciuc S, Bulone V. Quantitative Proteomic Analysis of Four Developmental Stages of *Saprolegnia parasitica*. Front Microbiol. 2018;8:2658.

10. Conesa A, Götz S. Blast2GO: A comprehensive suite for functional analysis in plant genomics. Int J Plant Genomics. 2008;2008:619832.

11. Marchler-Bauer A, Bo Y, Han L, He J, Lanczycki CJ, Lu S, et al. CDD/SPARCLE: functional classification of proteins via subfamily domain architectures. Nucleic Acids Res. 2016; 28(D1):200-203.
