# Supplementary material for: Comparison and optimization of protein extraction and two-dimensional gel electrophoresis protocols for liverworts
Source: BMC Res Notes. 2020 Feb 7;13:60. doi: 10.1186/s13104-020-4929-1 (PMC7006083; doi:10.1186/s13104-020-4929-1)
Supplement: Supplementary file 2 — Additional file 2. Additional results. [file 13104_2020_4929_MOESM2_ESM.docx]

**Additional file 2**

**Comparison and optimization of protein extraction and two-dimensional gel electrophoresis protocols for liverworts**

Sandhya Yadav^1^, Akanksha Srivastava^1^, Subhankar Biswas^1^, Neha Chaurasia^2^, Sushil Kumar Singh^3^, Sanjiv Kumar^4^, Vaibhav Srivastava^4^**^*^**, Yogesh Mishra^1^**^*^**

**Affiliations:**

*^1^Department of Botany, Centre of Advanced Study in Botany, Institute of Science, Banaras Hindu University, Varanasi-221005, India*

*^2^Department of Biotechnology and Bioinformatics, North Eastern Hill University,
Shillong- 793022, India*

*^3^Botanical Survey of India Northern Regional Centre, 192, Kaulagarh Road, Dehradun
Uttarakhand, 248003*, *India*

*^4^Division of Glycoscience, Department of Chemistry, School of Engineering Sciences in Chemistry, Biotechnology and Health, Royal Institute of Technology (KTH), AlbaNova University Centre, Stockholm,10691, Sweden*

***Corresponding Authors**: Vaibhav Srivastava (vasri@kth.se); Yogesh Mishra (ymishra@bhu.ac.in)

**Results**

**Comparison of the three different protein extraction buffers with respect to protein yield**

Three distinctive protein extraction followed by three protein precipitation protocols were employed on *D. hirsuta*, *M. paleacea*, and *P. appendiculatum*. The PVPP extraction buffer showed maximum protein yield in the crude extract of all three tested liverworts (Figure S1A). However, after protein precipitation in 20% TCA-acetone, the obtained results were strikingly different as 50 mM Tris-HCl (pH 7.5) buffer yielded more protein content compared to 1.5 M Tris-HCl (pH 8.8) and PVPP extraction buffer (Figure S1B).


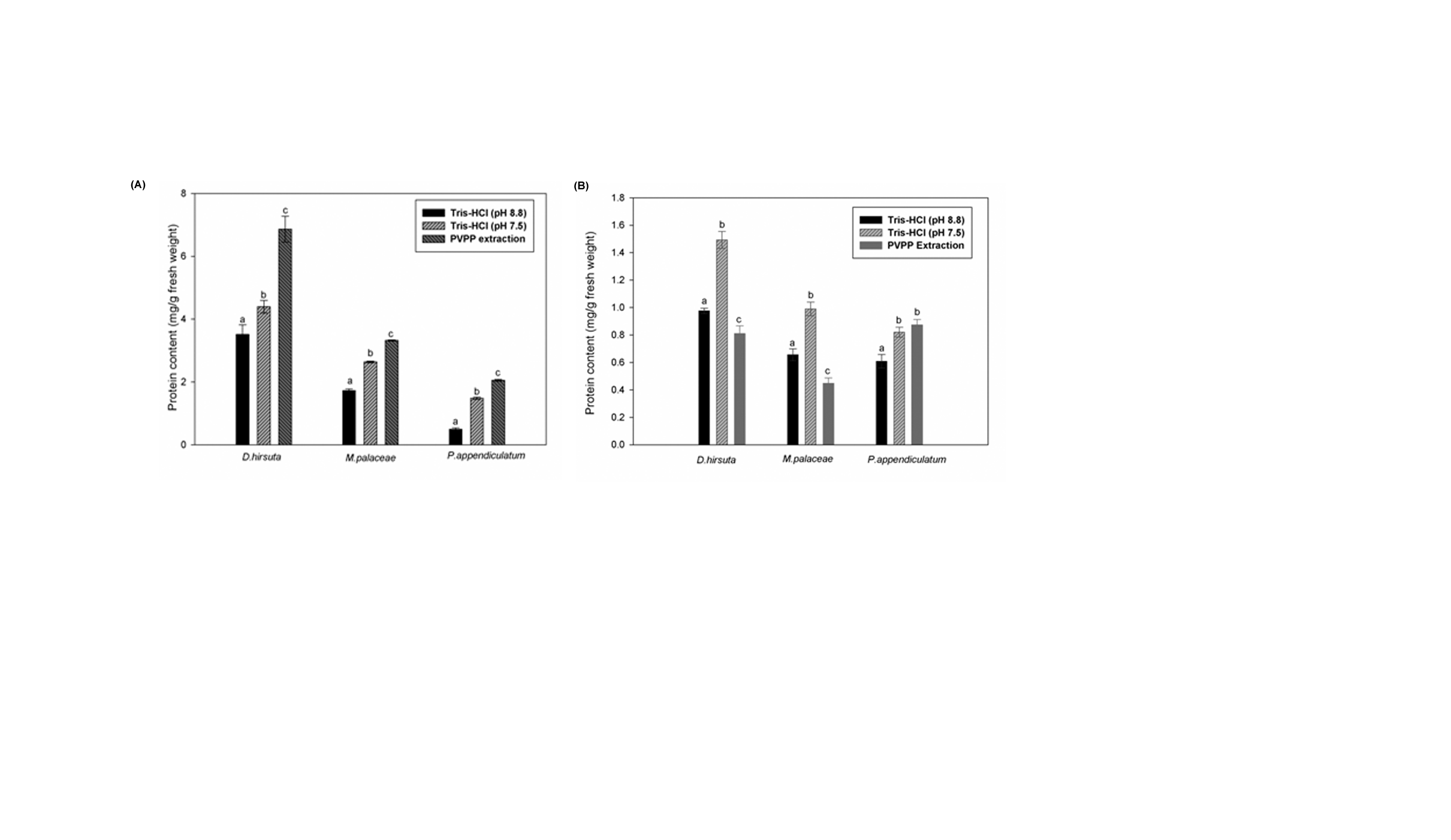


**Figure S1**. Evaluation of protein content (mg/g fresh weight) extracted by using buffers,namely,1.5 M Tris-HCl (pH 8.8), 50 mM Tris-HCl (pH 7.5), and PVPP extraction buffer **(A)** before **(B)** after 20% TCA-acetone precipitation in selected liverworts. Data are shown as the mean value (± S.D.), n=3.Values marked with letters are signiﬁcantly different from each other (p<0.05).

**50 mM Tris-HCl (pH 7.5) showed better results on SDS-PAGE for three tested liverworts**


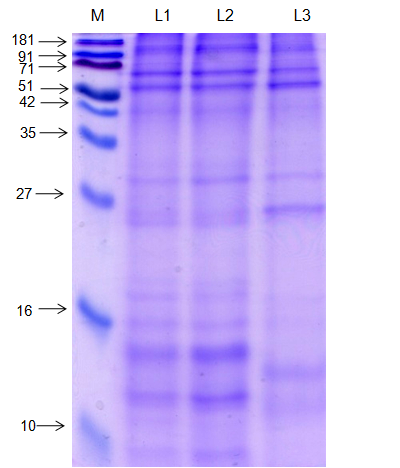
To examine the proteomic profiling of any organism at a preliminary level, it is important to start with one-dimensional gel electrophoresis, i.e. SDS-PAGE. We initially performed the SDS-PAGE profiling of *D. hirsuta* for the proteins extracted with 1.5 M Tris-HCl (pH 8.8), 50 mM Tris-HCl (pH 7.5), and PVPP extraction buffer to analyze optimal buffer giving better resolution and maximum protein bands. The results showed that proteins extracted using 50 mM Tris-HCl (pH 7.5) showed better band profile on SDS-PAGE compared to the remaining two buffers (Figure S2). Hence, 1.5 M Tris-HCl (pH 8.8) and PVPP extraction buffer was not considered for further studies.

**Figure S2**. SDS-PAGE profiling of total protein extracted from *D. hirsuta* using three different buffers: M- protein marker (kDa) and L1, L2 and L3-proteins extracted with 1.5 M Tris-HCl (pH 8.8), 50 mM Tris-HCl (pH 7.5), and PVPP extraction buffer, respectively.

**20% TCA-acetone aggressively removed the interfering compounds co-extracted with proteins and enhances the quality of 2-DE gels**

After having a preliminary idea of SDS-PAGE profile, we shifted to the next level, i.e. 2-DE. For performing 2-DE, protein sample is preferred in a precipitated form; therefore, we attempted different protein precipitation methods such as 80% ethanol, 80% acetone and TCA-acetone with varied concentration of TCA (10%, 15%, and 20%). We found that among these precipitation methods 20% TCA-acetone was more effective in case of removing non-protein interfering compounds because the resultant pellets were creamy white in colour rather than brown or yellow as the colour of precipitate attest the quality of protein [5] (Figure S3). In addition, protein sample with 20% TCA-acetone precipitation appeared to be better suitable method with higher spots number, spot intensity, and good resolution of 2-DE gels with no streaking and smearing compared to 80% ethanol, 80% acetone, 10 and 15% TCA-acetone (Figure S5 A-E). Further we applied this precipitation method along with rest of the two extraction buffers 1.5 M Tris-HCl (pH 8.8) and PVPP extraction buffer (Figure S4), where we found relatively dark coloured precipitate which was not suitable for 2-DE and has already been reported earlier [5].


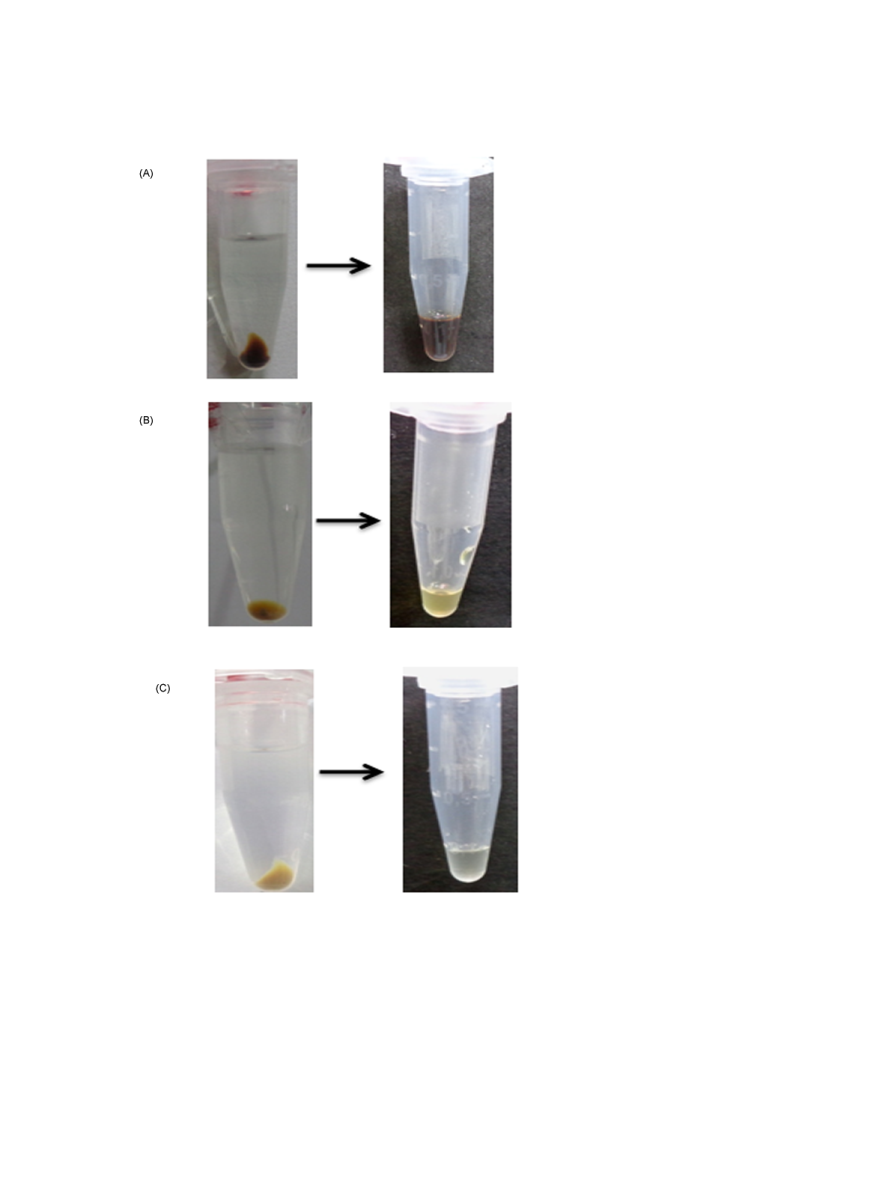


**
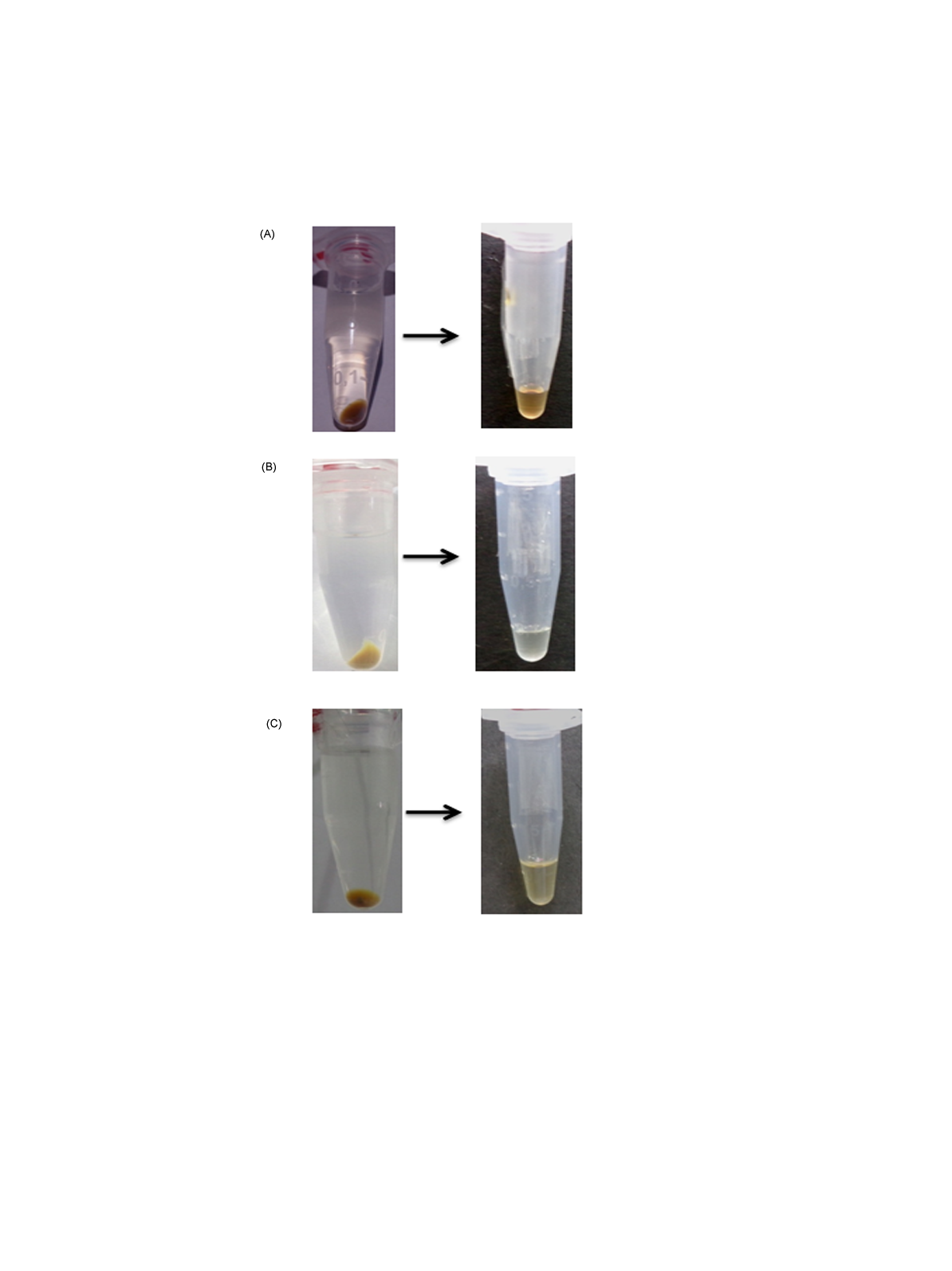
 Figure S3**.Protein pellets obtained after extraction in 50 mM Tris-HCl (pH 7.5) buffer followed by **(A)** 80% ethanol, **(B)** 80% acetone, **(C)** 20% TCA-acetone precipitation, followed by re-solubilization in rehydration buffer.

**Figure S4.** Protein pellets obtained after extraction in three selected buffers, followed by 20% TCA-acetone precipitation and re-solubilization in rehydration buffer (**A**) 1.5 M Tris-HCl (pH 8.8),(**B**) 50 mM Tris-HCl (pH 7.5), and (**C**) PVPP containing buffer.


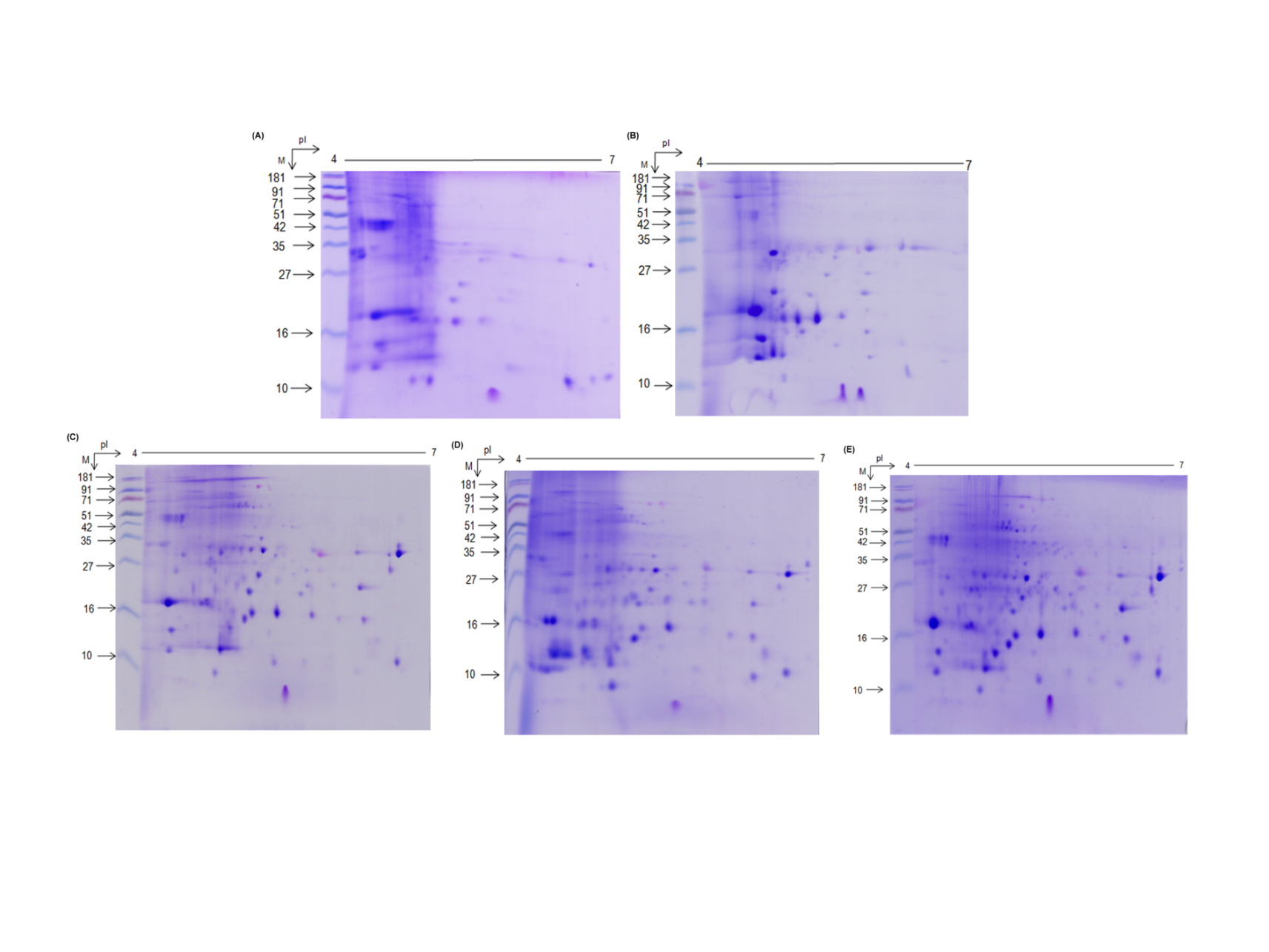


**Figure S5**. 2-DE gel images of *D. hirsuta* using **(A)** 80% ethanol, **(B)** 80% acetone **(C)** 10% TCA-acetone, **(D)** 15% TCA-acetone, and **(E)** 20% TCA-acetone precipitation.

**Sequential acetone concentration gradient washing of precipitated proteins improves the quality and solubility of proteins**

The resolution of 2-DE gels is significantly affected by the quality and solubility of proteins. Liverworts contain lots of secondary metabolites, which both affect the solubility of proteins and hamper the IEF. To improve the quality of proteins and to remove the contaminants co-precipitated with proteins, concentration gradient acetone washing of protein samples was done after thoroughly mixing the pellets using a micropipette. We noted that gradient acetone washing removed the contaminants and residual TCA by dissolving in it, which eventually enhanced the protein solubility in the rehydration buffer (data not shown).

**Prolonged incubation of protein pellets in rehydration buffer maximized protein solubilization**

Proteins obtained from TCA-acetone precipitation are sometimes challenging for complete resolubilization. Thus, to further enhance solubility, we increased the duration of protein solubilization in the rehydration buffer from 2 to 5 h at room temperature with occasional mixing using micropipette. This modified method of prolonged incubation in the rehydration buffer offered the maximum solubilization of proteins as tested by protein quantification (data are not shown).

**Slight alteration in IEF program enhanced the protein resolution and separation on 2-DE gel**

Initially, we followed the IEF program guidelines as provided in the instruction manual (GE-Healthcare) for running 13-cm IPG strips (500 V for 1 h, gradient of 1000 V for 1 h, gradient of 8000 V for 3 h, and 8000 V for 1 h). However, this program ensued in higher current in IEF instrument and poor separation on second dimension. To get rid of this problem, we added another step at the start of the IEF program (50 V for 3h). Starting of IEF at low voltage for long duration improved the sample entry that eventually enhanced the protein resolution on 2-DE gels (data are not shown).

**Two-fold increase in the SDS concentration and equilibration time enhanced the separation of high-molecular-weight proteins on 2-DE gel**

While performing 2-DE, we encountered a problem where high-molecular-weight proteins did not appear on the 2-DE gel (Figure S 5E/S6A). However, increase in the SDS concentration from normal 2% to 4% in the equilibration buffer and increase in the equilibration time from 15 min to 45 min removed the abovementioned problems and increased the appearance of high-molecular-weight protein spots on the gel (Figure S6B).


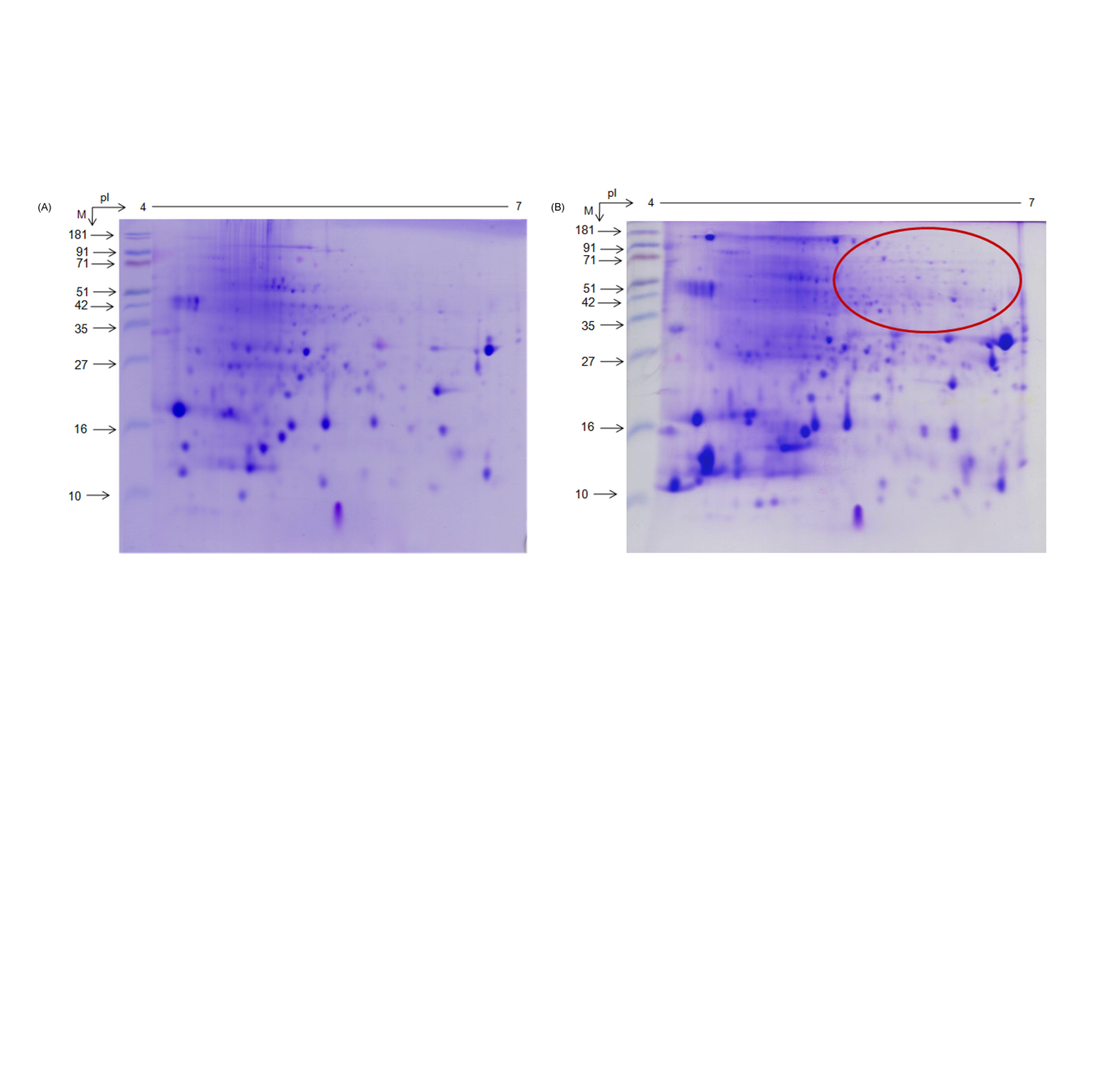


**Figure S6**. Comparison of proteome profile of *D. hirsuta* **(A)** with standard SDS concentration and an equilibration time of 15 min **(B)** with two-fold enhanced SDS concentration and an equilibration time from 15 min to 45 min.The red marked circles on 2-DE gels show new well-resolved spots in high-molecular-weight areas.

**Optimization of 2-DE for *D. hirsuta***

First, we optimized the 2-DE experiment in *D. hirsuta* using IPG strips (pH 3–10) of 13 cm for covering maximum proteins, and the result showed the distribution of spots across the gel; however, maximum spots approximately were confined in the region between pH 4 and 7 (Figure S7). Therefore, to further improve the resolution and enhance detection of low-resolved proteins, we performed all the remaining experiments in *D. hirsuta* using IPG strips of 13 cm (pH 4–7) (Figure S5 and S6).


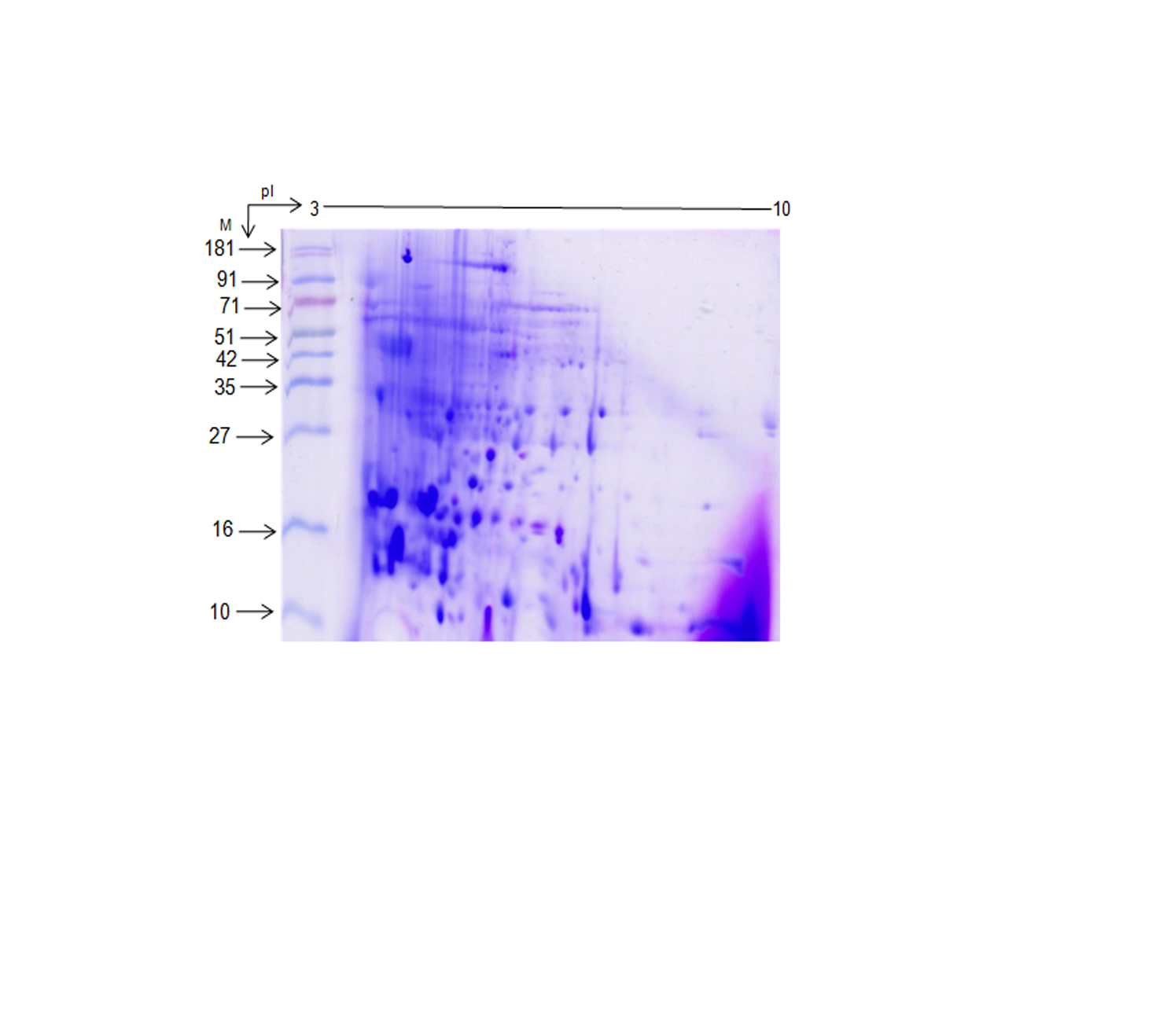


**Figure S7.** The 2-DE gel image of *D. hirsuta* with wider range of pH (3–10)

**Data analysis of 2-DE gels**

The 2***-***DE patterns of 50 mM Tris-HCl (pH 7.5) extracted proteins, followed by ice-chilled 80% ethanol, 80% acetone, and 20% TCA-acetone precipitation compared with equal amount of initial protein load, revealed that 20% TCA-acetone precipitation displayed a relatively better resolution with lesser contaminants, although proteins precipitated with 80% ethanol and 80% acetone resolved fewer protein spots along with vertical and horizontal streaking (Figure S 5 A and B). Note that we obtained ~376 detectable spots (as estimated by PDQuest software) by 20% TCA-acetone precipitated method, while only 65 and 87 spots were identified by 80% ethanol and 80% acetone, respectively. Fig. 9 shows the average number of spots from three replicate of 2-DE gels using 80% ethanol, 80% acetone, and 20% TCA-acetone precipitation method. We randomly selected 16 spots from the 20% TCA-acetone precipitated 2-DE gel of *D. hirsuta* for mass spectrometry (Figure S8).

**
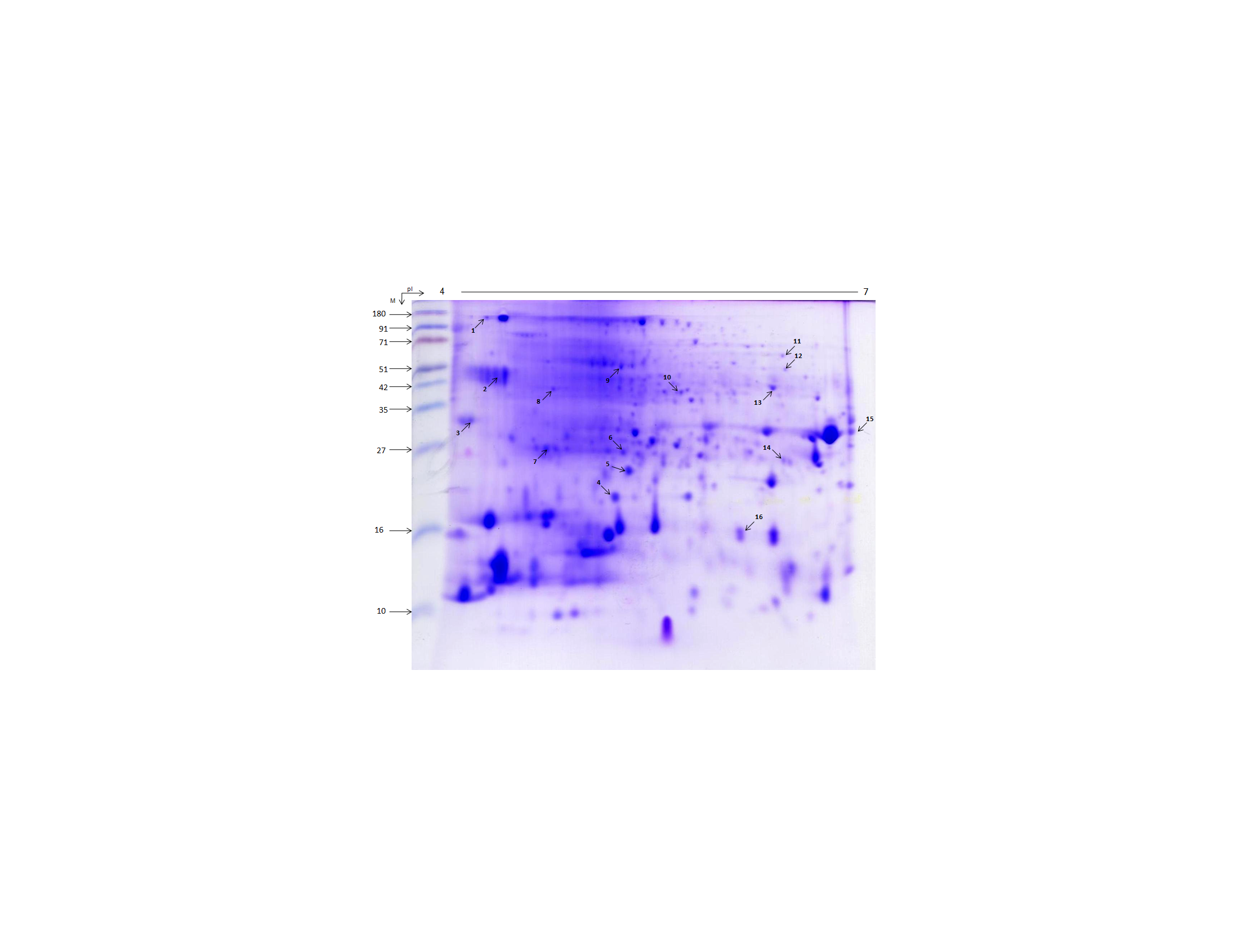
**

**Figure S8.** The marked arrows show the randomly selected spots for mass-spectrometry analysis from the 2-DE gel image of *D. hirsuta*.





**Figure S9.** Comparison of total number of protein spots appeared on 2-DE gels of *D. hirsuta* after using three different protein precipitation methods.

**Mass spectrometry for protein identification** All the 16 selected spots for mass spectrometry comprise both less abundant (1, 8, 11, 12, 14) and more abundant (2, 3, 4, 5, 6, 7, 9, 10, 13, 15, 16) proteins, which were successfully identified and listed in additional file 3:Table S1.

**Testing of phenol based method**


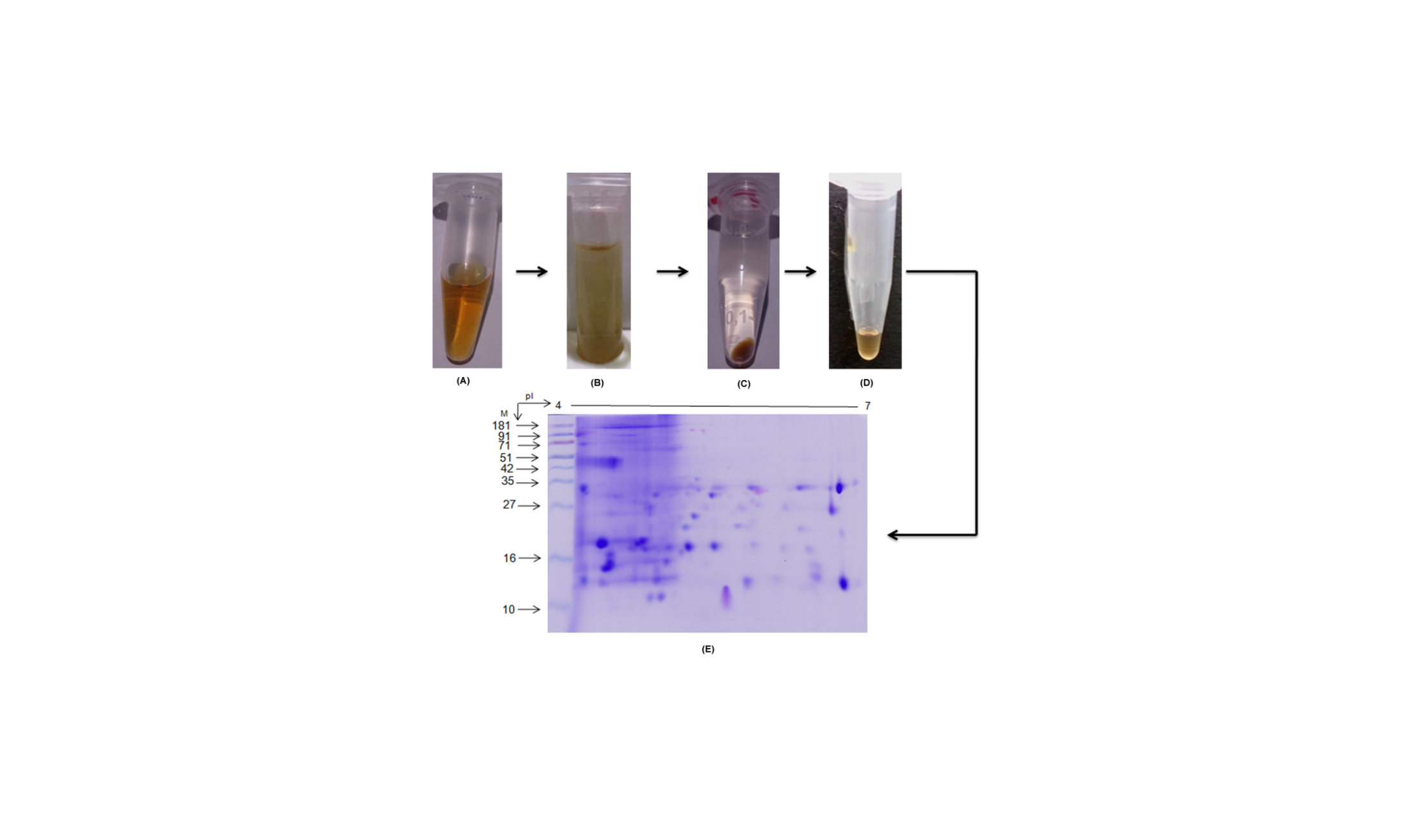
Apart from above mentioned protein extraction buffers, we also tested the well-known protein extraction method based on Tris-saturated phenol pH 7.5 [1] for *D. hirsuta* (Figure S10). However, this method was not very convincing because of less number of spots and low resolution of 2-DE gel therefore, it was not used for further proteomic study.

**Figure S10.** Image shows the steps of phenol extraction, followed by ammonium acetate-methanol precipitation in *D. hirsuta* (**A**), crude protein extract (**B**), precipitation in ammonium acetate-methanol (C) precipitated protein pellet (**D**) re-solubilized protein in rehydration buffer (**E**) 2-DE gel obtained using the above re-solubilized protein.

**Additional file 2 reference**

1. Wang X, Yang P, Gao Q, Liu X, Kuang T, Shen S, et al. Proteomic analysis of the response to high-salinity stress in *Physcomitrella patens*. Planta. 2008;228(1):167-177.
