# Supplementary material for: Comparison and optimization of protein extraction and two-dimensional gel electrophoresis protocols for liverworts
Source: BMC Res Notes. 2020 Feb 7;13:60. doi: 10.1186/s13104-020-4929-1 (PMC7006083; doi:10.1186/s13104-020-4929-1)
Supplement: Supplementary file 3 — Additional file 3: Table S1. List of identified proteins from the 2-DE gel of D. hirsuta using mass spectrometry. [file 13104_2020_4929_MOESM3_ESM.docx]

**Additional file 3**

**Table S1** List of identified proteins from the 2-DE gel of *D. hirsuta* using mass spectrometry

| **Spot No.** | **Accession No.** | **Description** | **No. of amino acids** | **Predicted mol. Wt. (kDa)** | **Observed mol. Wt. (kDa)** | **pI (predicted)** | **pI**  **(observed)** | **CDD(Conserved Domains)** | **No. of identified peptides** |
| --- | --- | --- | --- | --- | --- | --- | --- | --- | --- |
| 1 | PTQ32174.1 | HIPL1 protein | 779 | 83.947 | 94.9 | 6.5 | 4.5 | GSDH, Folate_rec | 4 |
| 2 | PTQ35949.1 | Class III  peroxidase | 346 | 36.59 | 45.0 | 4.3 | 4.6 | secretory_  peroxidase | 5 |
| 3 | PTQ35949.1 | Class III  peroxidase | 346 | 36.59 | 32.5 | 4.3 | 4.1 | Secretory_peroxidase | 2 |
| 4 | PTQ37565.1 | oxygen-evolving enhancer protein 1, chloroplastic | 329 | 33.98 | 25.7 | 4.8 | 5.2 | PLN00037 | 3 |
| 5 | PTQ47383.1 | psbP domain-containing protein 6, chloroplastic | 280 | 30.11 | 26.1 | 7.0 | 5.8 | DcrB | 2 |
| 6 | PTQ27665.1 | ascorbate peroxidase | 250 | 27.70 | 26.8 | 5.2 | 5.5 | ascorbate_peroxidase | 3 |
| 7 | PTQ28159.1 | type 1 glutamine amidotransferase domain-containing protein | 229 | 24.31 | 25.8 | 5.1 | 4.4 | GATase1_Hsp31_like | 8 |
| 8 | PTQ50374.1 | peptidyl-prolyl cis-trans isomerise CYP38, chloroplastic isoform X1 | 482 | 51.80 | 42.0 | 4.7 | 4.5 | cyclophilin_TLP40_like | 9 |
| 9 | PTQ34196.1 | ATP synthase subunit beta, mitochondrial-like | 562 | 60.31 | 57.0 | 6.4 | 5.3 | PRK09280 | 16 |
| 10 | PTQ27327.1 | malate dehydrogenase | 330 | 35.48 | 39.2 | 5.4 | 6.1 | PLN00135 | 3 |
| 11 | PTQ42371.1 | aldehyde dehydogenase family 2 member B7, mitochondrial-like | 545 | 59.03 | 65.5 | 7.3 | 6.8 | PLN02466 | 11 |
| 12 | PTQ37376.1 | monodehydroascorbate reductase 5, mitochondrial | 492 | 52.53 | 56.4 | 7.3 | 6.8 | Pyr_redox_2 | 12 |
| 13 | PTQ43664.1 | glycoside hydrolase | 408 | 46.58 | 41.5 | 5.8 | 6.6 | BglC | 9 |
| 14 | PTQ40055.1 | thioredoxin-like protein | 221 | 24.21 | 26.7 | 5.3 | 6.7 | PRX_1cys | 10 |
| 15 | PTQ36340.1 | peroxidise 12-like | 321 | 34.51 | 28.8 | 7.4 | 7.0 | plant_peroxidase_like | 2 |
| 16 | PTQ47922.1 | superoxide dismutase [Cu-Zn], chloroplastic-like | 161 | 16.37 | 15.0 | 6.0 | 6.4 | Cu-Zn_superoxide_Dismutase | 8 |
